# A prospective study on the changes and clinical significance of pre-operative and post-operative circulating tumor cells in resectable gastric cancer

**DOI:** 10.1186/s12967-018-1544-1

**Published:** 2018-06-20

**Authors:** Qiyue Zhang, Fei Shan, Ziyu Li, Jing Gao, Yilin Li, Lin Shen, Jiafu Ji, Ming Lu

**Affiliations:** 1 Key Laboratory of Carcinogenesis and Translational Research (Ministry of Education/Beijing), Department of GI Oncology, Peking University Cancer Hospital & Institute, Fucheng Road 52, Haidian District, Beijing, 100142 China; 2 Key Laboratory of Carcinogenesis and Translational Research (Ministry of Education/Beijing), Department of Surgery, Peking University Cancer Hospital & Institute, Fucheng Road 52, Haidian District, Beijing, 100142 China

**Keywords:** CellSearch, Circulating tumor cells, Hematogenous metastasis, Recurrence, Resectable gastric cancer

## Abstract

**Background:**

Circulating tumor cells (CTCs) have been suggested as potential prognostic indicators for multiple tumors, including gastric cancer; however, pre- and post-operative CTC changes in resectable gastric cancer and possible correlations to post-operative recurrence have not been evaluated.

**Methods:**

Subjects (n = 93) with resectable gastric cancer were prospectively reviewed from July 2013 to December 2014 at Peking University Cancer Hospital. The proportion of CTCs were evaluated before (n = 93) and after (n = 63) radical operation using a standardized CellSearch system.

**Results:**

CTCs ≥ 1 were measured in the pre-operative blood of 31 (33.3%) patients and in the post-operative blood of 21 patients (33.3%). Patients with relatively poor clinicopathological features had more pre- and post-operative CTCs. The 3-year disease-free survival (DFS) rate for patients with CTCs ≥ 5/7.5 ml was significantly lower than for patients with CTCs < 5/7.5 ml (40.0% vs 66.4%, p < 0.001 for pre-surgery; 25.0% vs 62.2%, p < 0.001 for post-surgery). Patients with CTCs ≥ 5/7.5 ml in post-operative blood had significantly shorter mean DFS (1.28 vs 31.6 months; p = 0.002) and overall survival (OS; 10.0 vs 34.9 months; p = 0.001) than other patients. Among the 10 patients with hematogenous recurrence, 3 had post-operative CTCs ≥ 2/7.5 ml and had early recurrence (DFS 1.1, 1.1, 1.4 months). Moreover, DFS for the seven patients was 20.2, 11.9, 20.0, 6.0, 15.5, 25.9, 30.0 months, respectively. DFS for the three patients with increased CTCs after surgery was shorter than for patients with mildly increased, stable, or decreased CTCs.

**Conclusions:**

Pre- and post-operative CTCs are promising prognostic markers for resectable gastric cancer. Our study further suggests that increased post-operative CTCs may be correlated with hematogenous recurrence.

*Trial registration* (ClinicalTrials.gov Identifier: NCT01848015). Registered 7 May 2013. https://clinicaltrials.gov/ct2/show/NCT01848015

**Electronic supplementary material:**

The online version of this article (10.1186/s12967-018-1544-1) contains supplementary material, which is available to authorized users.

## Background

Gastric cancer is a highly malignant disease, with high morbidity and mortality rates worldwide, especially in China [1]. Surgery is the most common curative approach for resectable gastric cancer; however, many patients, especially those with local advanced gastric cancer suffer from recurrence after radical gastrectomy, contributing to poor prognosis [2, 3]. Presently, poor clinicopathological characteristics (such as TNM classification, depth of tumor penetration, and lymph node metastasis) and high carcinoembryonic antigen (CEA) are predictive of high-risk post-operative tumor recurrence; but their use is limited due to low specificity and accuracy [4–7]. In addition to predicting patient prognosis, monitoring recurrence patterns of gastric cancer patients is vital. Hematogenous distant metastasis is a chief pattern of recurrence for gastric cancer patients. Yoo and colleagues confirmed that the mean time for hematogenous recurrence was the shortest [8]; however, TNM classification could not predict recurrence patterns of gastric cancer accurately. Therefore, the identification of effective markers is necessary to predict disease recurrence.

Circulating tumor cells (CTCs) are important markers of malignant tumor metastasis [9, 10] that can predict chemotherapeutic response and prognosis for multiple metastatic tumors [11–17]. In addition, pre- or post-operative CTCs have been reported to be correlated to post-operative recurrence of bladder, prostate, breast, and colorectal cancers, but data reported differ among studies [18–22]. Gazzaniga’s group reported that CTCs may predict decreased time to first tumor recurrence for stage I bladder cancer [21], but Pal and colleagues reported no correlation existed between CTCs and tumor recurrence for prostate cancer [22].

Correlations in pre- or post-operative CTCs to post-operation recurrence and prognosis for gastric cancer are also limited. Uenosono and colleagues analyzed CTCs in pre-operative peripheral blood samples of 148 patients who underwent gastrectomy, and intact CTCs in preoperative blood were significantly correlated with poor clinicopathological characteristics (depth of tumor invasion, lymph node metastasis, etc.) [23]. In addition, relapse-free and overall survival for patients with CTCs was significantly lower than for patients without CTCs (p < 0.001) [23]. Of note, pre-operative CTCs was an independent factor for overall survival (OS) for patients with gastric cancer according to multivariate analysis (p = 0.024). However, whether post-operative CTCs and changes in CTCs during surgery could be assessed for resectable gastric cancer (stage I–III) and any correlations with prognosis is unclear. Thus, we conducted this prospective study (ClinicalTrials.gov Identifier: NCT01848015), and measured CTCs in pre- and post-operative blood from patients with resectable gastric cancer and analyzed correlations of pre- and post-operative CTCs and further evaluated changes in CTCs during surgery with clinicopathological characteristics, prognosis, and recurrence patterns.

## Methods

### Patients and sample collection

From July 2013 to December 2014, 93 subjects with histologically confirmed gastric cancer who underwent a radical gastrectomy at the Department of Surgery in Peking University Cancer Hospital were enrolled. All of the patients were diagnosed with stage I–III gastric cancer. We excluded patients with distant metastases or those had undergone therapy prior to surgery, and 63 subjects had paired preoperative and postoperative blood samples. Peripheral blood was sampled within 1 week prior to surgery and post-operative samples were acquired a week after surgery. CTCs were counted for all of the subjects.

### Isolation and enumeration of circulating tumor cells

Circulating tumor cells were counted with a CellSearch system (Veridex, New Jersey, U.S.) according to previously published methods [17], which included a CellSearch Epithelial Cell Kit, CellPrep System, and CellSpotter Analyzer. In brief, 7.5 ml blood was drawn into 10-ml customized Cell Save Vacutainer tubes (Becton–Dickinson, New Jersey, U.S.), including EDTA and a cell fixative, followed by CTC enumeration within 72 h. Reagents used included anti-epithelial cell adhesion molecule (anti-EpCAM) antibody-coated magnetic beads, fluorescent dye-labeled anti-CD45 antibody and anti-cytokeratins (CK-8, -18, and -19) antibody, and 4′, 6-diamidino-2-phenylindole (DAPI). CTCs identified with the CellSearch system were CK-positive, DAPI-positive, and CD45-negative.

### Statistical analysis and illustrations

Statistical analysis was performed using SPSS 18.0 software (SPSS Inc., Chicago, IL). Correlations of CTCs to clinicopathological characteristics were analyzed with Pearson’s Chi Square or Fisher’s exact test. Disease-free survival (DFS) was defined from the date of surgery to post-operative recurrence or death caused by any reason. Overall survival (OS) was defined from the date of surgery to death caused by any reason. Correlations of pre- and postoperative CTC counts to DFS or OS were analyzed using Kaplan–Meier tests. Crosstab was used to analyze the relationship between 3-year disease-free survival rate (3-year DFS) and CTCs in pre- and post-surgical blood or clinicopathological characteristics. A two-sided p < 0.05 was considered to be statistically significant. All of the illustrations were created by Photoshop CS4 (Adobe).

## Results

### Patient characteristics

Patients (n = 93) with resectable gastric cancer were prospectively assessed. Patient characteristics are presented in Table 1. In total, 68 male and 25 female patients were included in this study, with the median age of 60 years (26–82 years). Twenty-nine (31.2%) patients had primary tumors located in gastroesophageal junction, and 71 patients (76.3%) had tumors with poor differentiation (including low differentiation, mucinous adenocarcinoma, and signet-ring cell carcinoma). The number of patients with stage I, II, and III were 24 (25.8%), 24 (25.8%), and 45 (48.4%), respectively.

**Table 1:** Characteristics of patients

Characteristics	Pre-operation (n = 93)	Post-operation (n = 63)
	No. of Patients (%)	No. of Patients (%)
Gender		
Male	68 (73.1%)	46 (73.0%)
Female	25 (26.9%)	17 (27.0%)
Age (years)		
≤ 45	16 (17.2%)	13 (20.6%)
> 45	77 (82.8%)	50 (79.4%)
Primary site^a^		
EGJ	29 (31.2%)	19 (30.2%)
Non-EGJ	64 (68.8%)	44 (69.8%)
Differentiation^b^		
Good	22 (23.7%)	15 (23.8%)
Poor	71 (76.3%)	48 (76.2%)
Lauren		
Intestinal	36 (38.7%)	25 (39.7%)
Diffuse	23 (24.7%)	16 (25.4%)
Mixed	34 (36.6%)	22 (34.9%)
TNM classification		
I	24 (25.8%)	15 (23.8%)
II	24 (25.8%)	16 (25.4%)
III	45 (48.4%)	32 (50.8%)
Depth of penetration		
T1/T2	30 (32.3%)	19 (30.2%)
T3	34 (36.6%)	25 (39.7%)
T4	29 (31.2%)	19 (30.2%)
Lymph node		
N0	31 (33.3%)	16 (25.4%)
N1/N2	31 (33.3%)	26 (41.3%)
N3	31 (33.3%)	21 (33.3%)
Lymph-vascular invasion		
Yes	45 (48.4%)	34 (54.0%)
No	48 (51.6%)	29 (46.0%)
CEA^c^ level before operation (ng/ml)		
< 5	75 (80.6%)	51 (81.0%)
≥ 5	17 (18.3%)	12 (19.0%)
NA^d^	1 (1.1%)	0 (0.0%)
Adjuvant chemotherapy		
Yes	56 (60.2%)	42 (66.7%)
No	37 (39.8%)	21 (33.3%)

^a^EGJ, gastroesophageal junction

^b^Good, including high or moderate differentiation; Poor, including low differentiation, mucinous adenocarcinoma, and signet-ring cell carcinoma

^c^carcinoembryonic antigen

^d^non-available

### Correlations of CTCs to clinicopathological characteristics

**Fig. 1:**
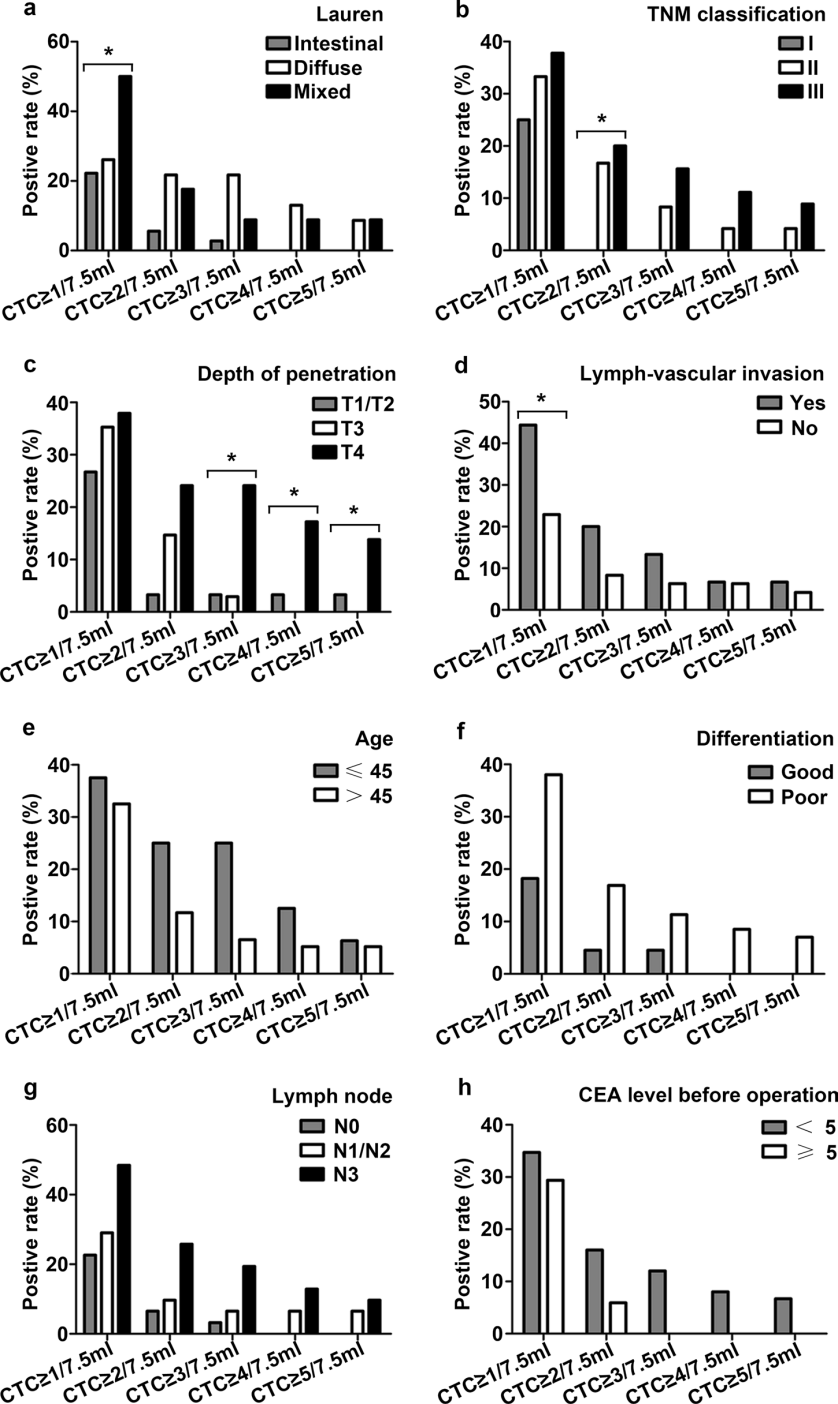
CTCs were significantly (p < 0.05) correlated with Lauren (**a**), TNM classification (**b**), depth of penetration (**c**) and lymph-vascular invasion (**d**), and were obviously, though not significantly, correlated with age (**e**), differentiation (**f**), lymph node metastasis (**g**), and CEA level (**h**) in preoperative blood, *p < 0.05

**Fig. 2:**
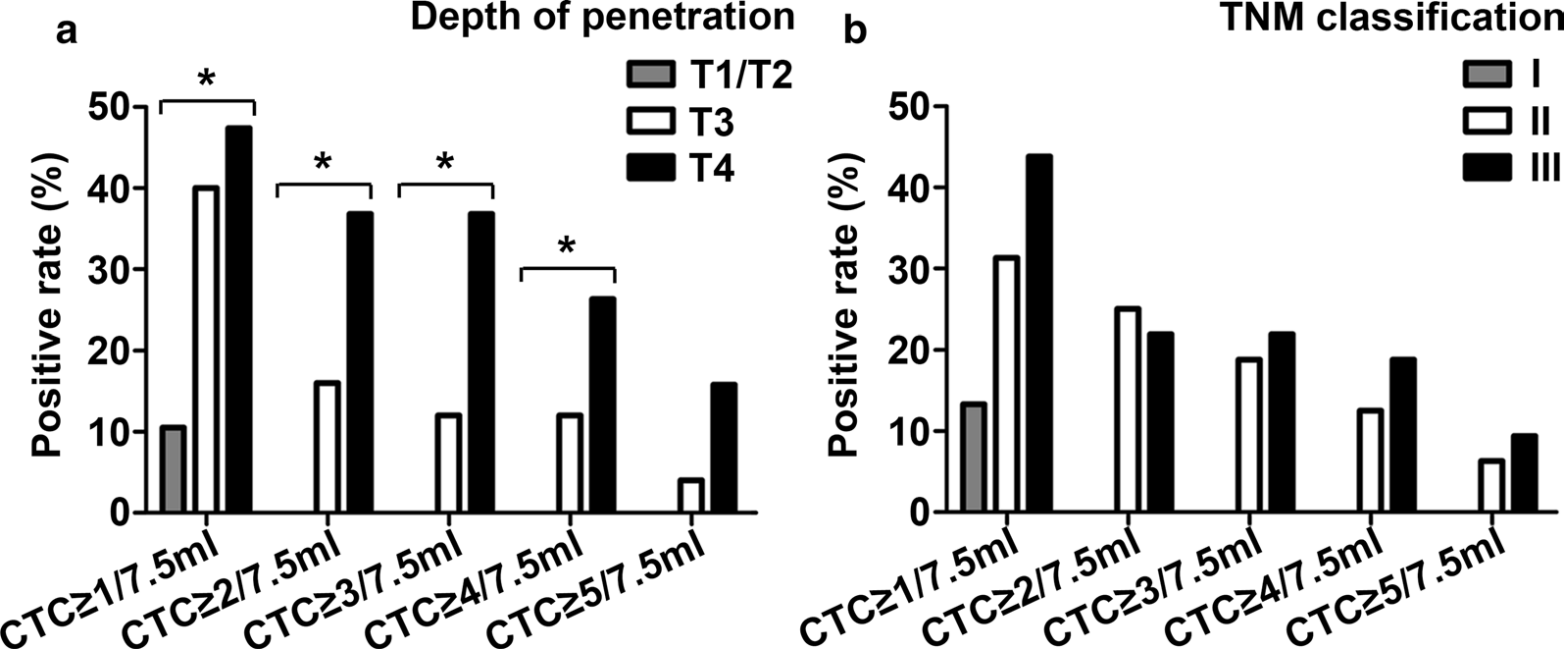
CTCs were significantly (p < 0.05) correlated with depth of penetration (**a**) and were obviously, though not significantly, correlated with TNM classification (**b**) in postoperative blood, *p < 0.05

For preoperation blood collection, ≥ 1, ≥ 2, ≥ 3, ≥ 4, and ≥ 5 CTCs/7.5 ml were found in 31 (33.3%), 13 (14.0%), 9 (9.7%), 6 (6.5%), and 5 (5.4%) patients, respectively. In addition, for postoperation blood collection, ≥ 1, ≥ 2, ≥ 3, ≥ 4, and ≥ 5 CTCs/7.5 ml were found in 21 (33.3%), 11 (17.5%), 10 (15.9%), 8 (12.7%), and 4 (6.3%) patients, respectively. Preoperative CTCs data are presented in Table 2 and Fig. 1. CTCs were significantly (p < 0.05) correlated with Lauren, TNM classification, depth of penetration, and lymph-vascular invasion (Fig. 1a–d). However, there was no significant (p > 0.05) correlation between CTCs and age, differentiation, lymph node metastasis, and CEA levels prior to surgery (Fig. 1e–h). Moreover, patients no older than 45 years-of-age, with low differentiation and lymph node metastasis were more likely to have more CTCs. Additional file 1: Table S1 depicts patients CTCs data pre- and post-surgery. Post-operative CTCs were correlated with patient characteristics in a manner similar to pre-operative CTCs (Table 3). Patients with relatively poor clinicopathological features had more post-operative CTCs, and this was significantly correlated with depth of penetration (p < 0.05) and non-significantly correlated with TNM classification (p > 0.05; Fig. 2). Ten patients had ≥ 3 CTCs/7.5 ml in post-operative samples and most had poor differentiation (n = 8), stage IIB/III (n = 10), or T3/T4 (n = 10) penetration.

**Table 2:** Correlation of preoperative CTC number to clinicopathological characteristics

Characteristics	3-year recurrence rate%	No. of patients (%)				
		CTC ≥ 1/7.5 ml	CTC ≥ 2/7.5 ml	CTC ≥ 3/7.5 ml	CTC ≥ 4/7.5 ml	CTC ≥ 5/7.5 ml
Gender						
Male (n = 68)	65.6	23 (33.8%)	9 (13.2%)	6 (8.8%)	5 (7.4%)	5 (7.4%)
Female (n = 25)	63.1	8 (32.0%)	4 (16.0%)	3 (12.0%)	1 (4.0%)	0 (0.0%)
*p* value	0.658	0.869	0.997	0.949	1.000	0.319
Age						
≤ 45 (n = 16)	58.3	6 (37.5%)	4 (25.0%)	4 (25.0%)	2 (12.5%)	1 (6.3%)
> 45 (n = 77)	66.2	25 (32.5%)	9 (11.7%)	5 (6.5%)	4 (5.2%)	4 (5.2%)
*p* value	0.244	0.698	0.317	0.070	0.274	1.000
Primary site^a^						
EGJ (n = 29)	50.4	9 (31.0%)	3 (10.3%)	2 (6.9%)	2 (6.9%)	2 (6.9%)
Non-EGJ (n = 64)	71.2	22 (34.3%)	10 (15.6%)	7 (10.9%)	4 (6.3%)	3 (4.7%)
*p* value	*0.002*	0.873	0.787	0.873	1.000	0.635
Differentiation^b^						
Good (n = 22)	71.8	4 (18.2%)	1 (4.5%)	1 (4.5%)	0 (0.0%)	0 (0.0%)
Poor (n = 71)	62.6	27 (38.0%)	12 (16.9%)	8 (11.3%)	6 (8.5%)	5 (7.0%)
*p* value	0.174	0.084	0.268	0.604	0.330	0.335
Lauren						
Intestinal (n = 36)	68.0	8 (22.2%)	2 (5.6%)	1 (2.8%)	0 (0.0%)	0 (0.0%)
Diffuse (n = 23)	67.4	6 (26.1%)	5 (21.7%)	5 (21.7%)	3 (13.0%)	2 (8.7%)
Mixed (n = 34)	60.0	17 (50.0%)	6 (17.6%)	3 (8.8%)	3 (8.8%)	3 (8.8%)
*p* value	0.434	*0.033*	0.118	0.060	0.068	0.148
TNM classification						
I (n = 24)	100.0	6 (25.0%)	0 (0.0%)	0 (0.0%)	0 (0.0%)	0 (0.0%)
II (n = 24)	68.3	8 (33.3%)	4 (16.7%)	2 (8.3%)	1 (4.2%)	1 (4.2%)
III (n = 45)	44.0	17 (37.8%)	9 (20.0%)	7 (15.6%)	5 (11.1%)	4 (8.9%)
*p* value	*0.000*	0.563	*0.047*	0.103	0.306	0.443
Depth of penetration						
T1/T2 (n = 30)	90.0	8 (26.7%)	1 (3.3%)	1 (3.3%)	1 (3.3%)	1 (3.3%)
T3 (n = 34)	56.9	12 (35.3%)	5 (14.7%)	1 (2.9%)	0 (0.0%)	0 (0.0%)
T4 (n = 29)	47.7	11 (37.9%)	7 (24.1%)	7 (24.1%)	5 (17.2%)	4 (13.8%)
*p* value	*0.000*	0.627	0.064	*0.007*	*0.009*	*0.026*
Lymph node						
N0 (n = 31)	96.8	7 (22.6%)	2 (6.5%)	1 (3.2%)	0 (0.0%)	0 (0.0%)
N1/N2 (n = 31)	58.4	9 (29.0%)	3 (9.7%)	2 (6.5%)	2 (6.5%)	2 (6.5%)
N3 (n = 31)	38.9	15 (48.4%)	8 (25.8%)	6 (19.4%)	4 (12.9%)	3 (9.7%)
*p* value	*0.000*	0.081	0.109	0.133	0.160	0.362
Lymph-vascular invasion						
Yes (n = 45)	53.0	20 (44.4%)	9 (20.0%)	6 (13.3%)	3 (6.7%)	3 (6.7%)
No (n = 48)	75.8	11 (22.9%)	4 (8.3%)	3 (6.3%)	3 (6.3%)	2 (4.2%)
*p* value	*0.001*	*0.028*	0.105	0.307	1.000	0.671
CEA^c^ level before operation (ng/ml)						
< 5 (n = 75)	68.8	26 (34.7%)	12 (16.0%)	9 (12.0%)	6 (8.0%)	5 (6.7%)
≥ 5 (n = 17)	46.3	5 (29.4%)	1 (5.9%)	0 (0.0%)	0 (0.0%)	0 (0.0%)
NA^d^ (n = 1)		0 (0.0%)	0 (0.0%)	0 (0.0%)	0 (0.0%)	0 (0.0%)
*P* value	*0.001*	0.679	0.487	0.293	0.589	0.580

Italic values indicate that there are significant differences in pre-operative CTCs between different clinicopathological types

^a^EGJ, gastroesophageal junction

^b^Good, including high or moderate differentiation; Poor, including low differentiation, mucinous adenocarcinoma, and signet-ring cell carcinoma

^c^carcinoembryonic antigen

^d^non-available

**Table 3:** Correlation of postoperative CTC number to clinicopathological characteristics

Characteristics	3-year recurrence rate%	No. of patients (%)				
		CTC ≥ 1/7.5 ml	CTC ≥ 2/7.5 ml	CTC ≥ 3/7.5 ml	CTC ≥ 4/7.5 ml	CTC ≥ 5/7.5 ml
Gender						
Male (n = 46)	60.9	18 (39.1%)	10 (21.7%)	9 (19.6%)	7 (15.2%)	3 (6.5%)
Female (n = 17)	57.5	3 (17.6%)	1 (5.9%)	1 (5.9%)	1 (5.9%)	1 (5.9%)
*p* value	0.606	0.108	0.272	0.352	0.574	1.000
Age						
≤ 45 (n = 13)	57.1	5 (38.5%)	2 (15.4%)	2 (15.4%)	2 (15.4%)	1 (7.7%)
> 45 (n = 50)	60.6	16 (32.0%)	9 (18.0%)	8 (16.0%)	6 (12.0%)	3 (6.0%)
*p* value	0.565	0.912	1.000	1.000	1.000	1.000
Primary site^a^						
EGJ (n = 19)	43.5	7 (36.8%)	3 (15.8%)	3 (15.8%)	3 (15.8%)	1 (5.3%)
Non-EGJ (n = 44)	67.2	14 (31.8%)	8 (18.2%)	7 (15.9%)	5 (11.4%)	3 (6.8%)
*p* value	*0.001*	0.698	1.000	1.000	0.943	1.000
Differentiation^b^						
Good (n = 15)	66.0	5 (33.3%)	3 (20.0%)	2 (13.3%)	1 (6.7%)	0 (0.0%)
Poor (n = 48)	57.8	16 (33.3%)	8 (16.7%)	8 (16.7%)	7 (14.6%)	4 (8.3%)
*p* value	0.244	1.000	1.000	1.000	0.719	0.564
Lauren						
Intestinal (n = 25)	62.0	8 (32.0%)	5 (20.0%)	4 (16.0%)	2 (8.0%)	0 (0.0%)
Diffuse (n = 16)	65.7	4 (25.0%)	3 (18.8%)	3 (18.8%)	3 (18.8%)	2 (12.5%)
Mixed (n = 22)	52.4	9 (40.9%)	3 (13.6%)	3 (13.6%)	3 (13.6%)	2 (9.1%)
*p* value	0.115	0.580	0.844	0.913	0.505	0.167
TNM classification						
I (n = 15)	100.0	2 (13.3%)	0 (0.0%)	0 (0.0%)	0 (0.0%)	0 (0.0%)
II (n = 16)	54.7	5 (31.3%)	4 (25.0%)	3 (18.8%)	2 (12.5%)	1 (6.3%)
III (n = 32)	43.1	14 (43.8%)	7 (21.9%)	7 (21.9%)	6 (18.8%)	3 (9.4%)
*p* value	0.062	0.117	0.106	0.161	0.249	0.798
Depth of penetration						
T1/T2 (n = 19)	89.5	2 (10.5%)	0 (0.0%)	0 (0.0%)	0 (0.0%)	0 (0.0%)
T3 (n = 25)	51.2	10 (40.0%)	4 (16.0%)	3 (12.0%)	3 (12.0%)	1 (4.0%)
T4 (n = 19)	41.4	9 (47.4%)	7 (36.8%)	7 (36.8%)	5 (26.3%)	3 (15.8%)
*p* value	*0.000*	*0.036*	*0.008*	*0.005*	*0.043*	0.178
Lymph node						
N0 (n = 16)	100.0	4 (25.0%)	2 (12.5%)	1 (6.3%)	0 (0.0%)	0 (0.0%)
N1/N2 (n = 26)	54.5	8 (30.8%)	6 (23.1%)	6 (23.1%)	5 (19.2%)	2 (7.7%)
N3 (n = 21)	33.3	9 (42.9%)	3 (14.3%)	3 (14.3%)	3 (14.3%)	2 (9.5%)
*p* value	*0.001*	0.488	0.761	0.375	0.185	0.663
Lymph-vascular invasion						
Yes (n = 34)	49.8	10 (29.4%)	5 (14.7%)	5 (14.7%)	3 (8.8%)	2 (5.9%)
No (n = 29)	71.4	11 (37.9%)	6 (20.7%)	5 (17.2%)	5 (17.2%)	2 (6.9%)
*p* value	*0.002*	0.475	0.533	1.000	0.453	1.000
CEA^c^ level before operation (ng/ml)						
< 5 (n = 51)	62.6	18 (35.3%)	9 (17.6%)	9 (17.6%)	8 (15.7%)	4 (7.8%)
≥ 5 (n = 12)	48.6	3 (25.0%)	2 (16.7%)	1 (8.3%)	0 (0.0%)	0 (0.0%)
*p* value	*0.046*	0.734	1.000	0.722	0.324	1.000

Italic values indicate that there are significant differences in post-operative CTCs between different clinicopathological types

^a^EGJ, gastroesophageal junction

^b^Good, including high or moderate differentiation; Poor, including low differentiation, mucinous adenocarcinoma, and signet-ring cell carcinoma

^c^carcinoembryonic antigen

### Correlations of CTCs enumeration with DFS and OS in resectable gastric cancer

**Fig. 3:**
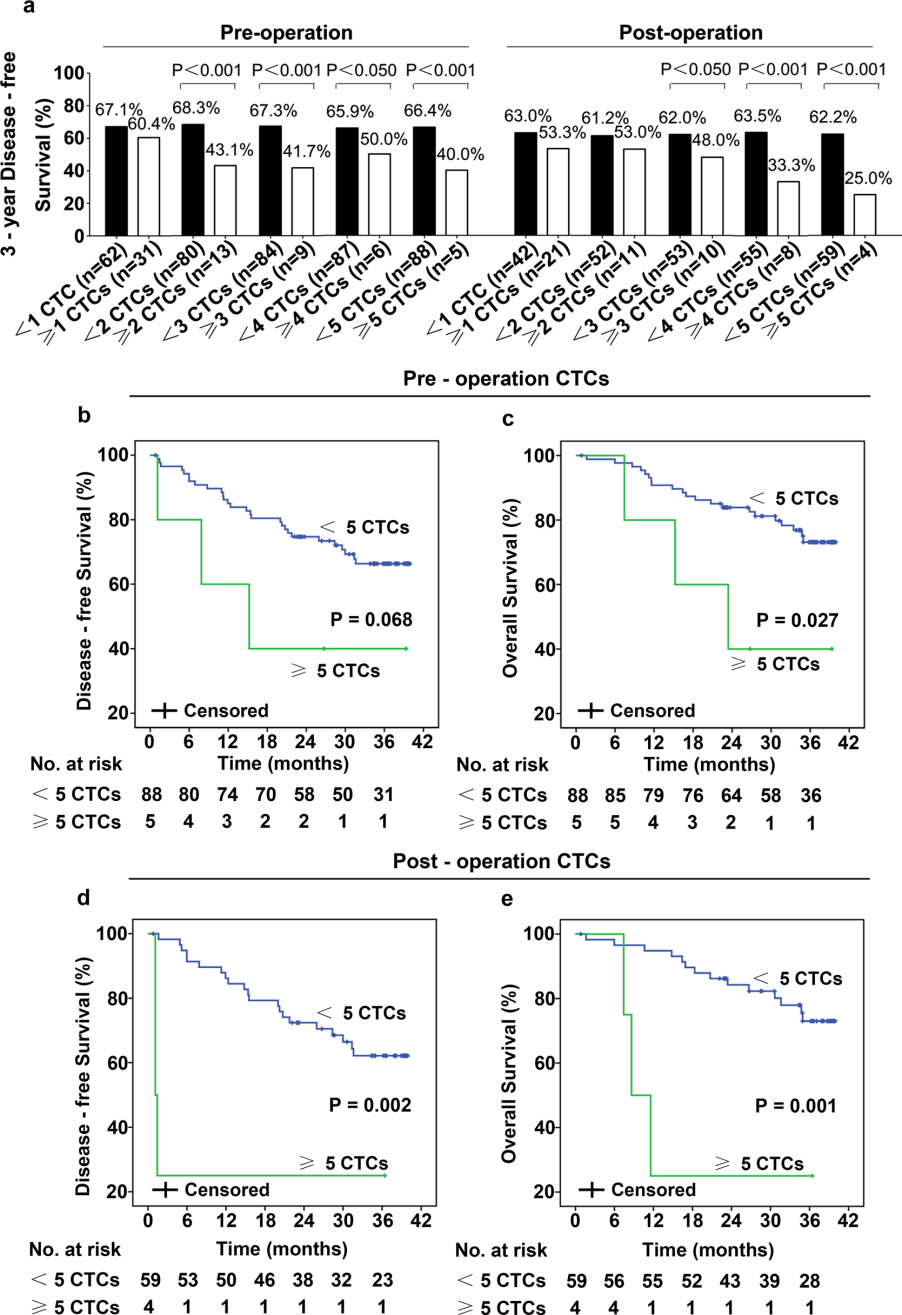
3-year DFS was lower in patients who had more CTCs (**a**), the filled black columns represent the 3-year DFS of patients who had CTCs no more than the cut-off value, and the empty white columns represent 3-year DFS of patients who had CTC more than the cutoff value. Patients who have ≥ 5 CTCs/7.5 ml blood pre-operatively had shorter DFS (**b**, **d**) and OS (**c**, **e**)

Until November 2016, median follow-up was 36.4 [interquartile 33.9–38.7] months. In total, 31 (33.3%) patients relapsed, 24 (25.8%) patients died. Patients CTC and DFS data are presented in Additional file 2: Table S2. In our study, patients with gastroesophageal junction, T3/T4 penetration, lymph node metastases, CEA level ≥ 5 ng/ml and higher pre- or postoperative CTCs had lower 3-year DFS (Table 2 and Fig. 3a). The 3-year DFS was 100.0% in patients with stage I tumors, 68.3% in patients with stage II tumors, 44.0% in patients with stage III tumors (Table 2). As shown in Fig. 3a, patients who had more CTCs had lower 3-year DFS. Obviously, as the threshold increased, the 3-year DFS of patients with post-operative CTCs above each cut-off value decreased markedly. Moreover, 3-year DFS in patients with CTCs ≥ 5/7.5 ml was significantly lower than in patients with CTCs < 5/7.5 ml (40.0% vs 66.4%, p < 0.001 for preoperation CTCs; 25.0% vs 62.2%, p < 0.001 for postoperation CTCs; Fig. 3a). In addition, patients with ≥ 5 CTCs/7.5 ml blood pre-operatively had shorter DFS (p = 0.068) and OS (p = 0.027) as assessed by univariate analyses (Fig. 3b, c). Likewise, patients with CTCs ≥ 5/7.5 ml in postoperative blood had significantly shorter DFS (1.28 vs 31.6 months; p = 0.002) and OS (10.0 vs 34.9 months; p = 0.001) than patients with CTCs < 5/7.5 ml (Fig. 3d, e). These results indicated that postoperative CTCs may be an effective prognostic marker in resectable gastric cancer; therefore, more attention and proper adjuvant chemotherapy should be given to patients who have higher post-operative CTCs.

### Changes of CTCs during surgery to recurrence pattern

**Fig. 4:**
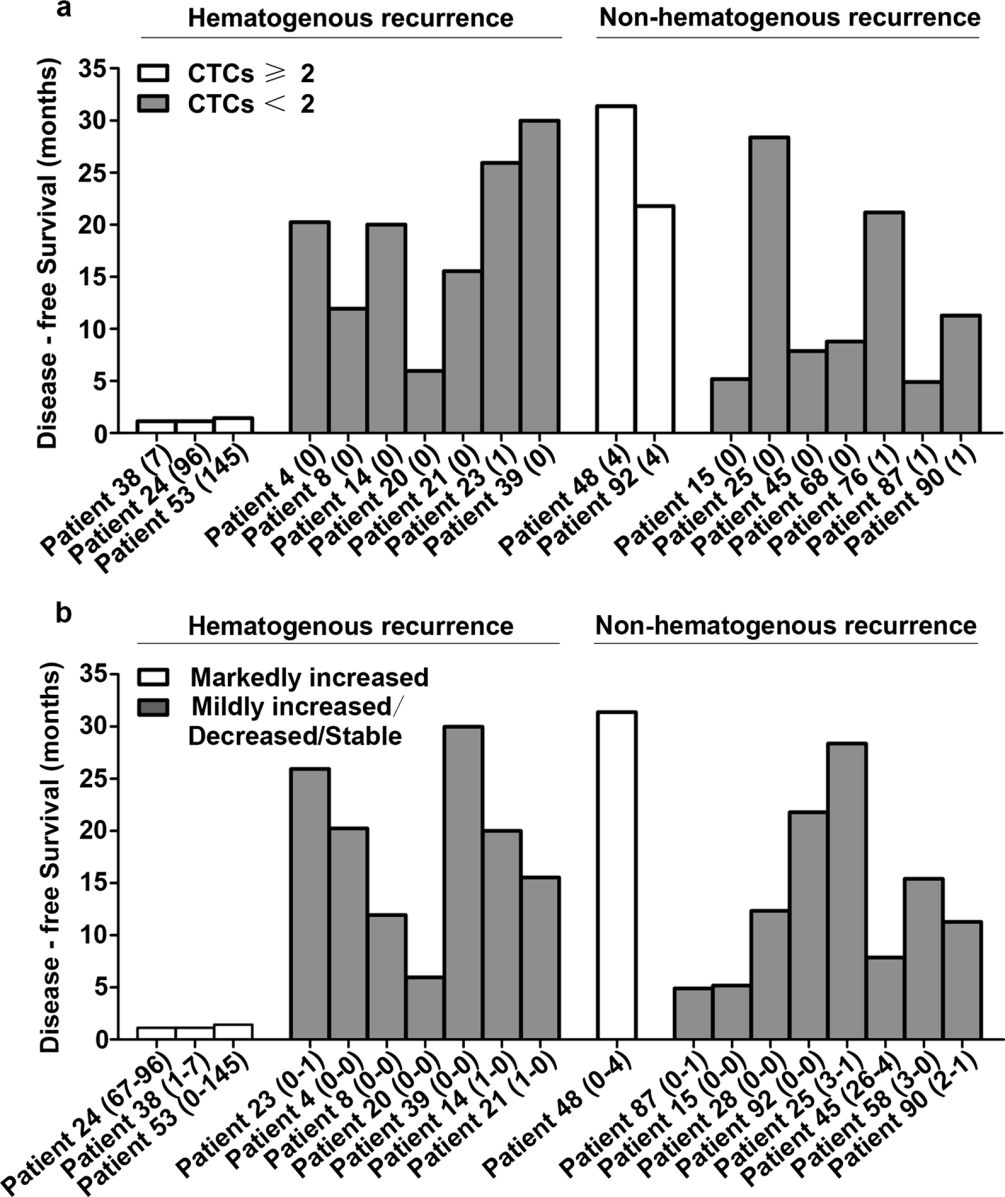
Among hematogenous metastases subjects, patients who had post-operative CTCs ≥ 2/7.5 ml (**a**) and patients who had markedly increased CTCs after surgery (**b**) had markedly shorter DFS

Among the 31 patients who relapsed, 10 patients had hematogenous (osseous, hepatic and lung) metastases, and 14 patients had non-hematogenous (peritoneal and locoregional) metastases. The remaining seven patients had no identified hematogenous recurrence or non-hematogenous recurrence until death. We found that pre-operative CTCs were not correlated with the recurrence pattern (Additional file 3: Figure S1). Patients with hematogenous metastases who had post-operative CTCs ≥ 2/7.5 ml had markedly shorter DFS (Fig. 4a). The DFS of three patients with hematogenous metastases who had markedly increased CTCs after surgery was short (1.1, 1.1, and 1.4 months, respectively, Fig. 4b); however, the DFS of other patients was relatively longer (25.9, 20.2, 11.9, 6.0, 30.0, 20.0 and 15.5 months, respectively). For patients with non-hematogenous metastases, the post-operative CTCs and changes of CTCs during surgery were not correlated with DFS. Post-operative CTC and markedly increased CTC during surgery may have potential association with hematogenous metastasis and suggest early recurrence.

## Discussion

Surgery is the first-line therapy for gastric cancer, especially for patients with resectable gastric cancer; however, tumor recurrence within 5 years suggests a poor prognosis [2]. The 5-year DFS for patients with stage II, IIIA, and IIIB gastric cancer are 64.4, 50.0, and 34.4%, respectively [2]. Poor clinicopathological features and high CEA and CA-19-9 tumor markers are risk factors for post-operative tumor recurrence, even though these markers have limited clinical significance [4–7]. Thus, there is a need to distinguish patients with high post-operative recurrence risk with effective biomarkers.

Recently, CTCs have been suggested to have utility as a predictor of chemotherapeutic response and prognosis, as CTCs can predict relapse-free survival in metastatic colorectal, prostate, and breast cancers [11–16]. For resectable gastric cancer, only one study reported that relapse-free survival was less common for patients with measurable pre-operative CTCs compared to those with non-detectable CTCs [23]. Therefore, we measured pre- and post-operative CTCs and changes in CTCs during surgery in patients with resectable gastric cancer and correlated these changes with clinicopathological features, post-operative tumor recurrence, and recurrence patterns.

Circulating tumor cells measured in 31 patients prior to surgery were greater than those reported in another study using a prospective cohort [23]. Differences in clinicopathological features between our and other cohorts may explain this discrepancy, such as differences in TNM classification and depth of tumor penetration. In our study, 63 patients had paired pre- and post-operative samples and CTCs were found in 21 patients post-operatively. These data demonstrated that CTCs may still appear in circulation after radical surgery. Thus, long-term monitoring CTCs after surgery may predict post-operative tumor recurrence.

Patients with relatively poor clinicopathological features had greater pre- and post-operative CTCs. Poor clinicopathological features were correlated with high post-operative tumor recurrence risk and poor prognosis, suggesting that patients with higher pre- or post-operative CTCs had earlier recurrence. Until now, studies of correlations in CTCs with recurrence of resectable gastric cancer have been limited, so there is no standard threshold. To address this issue, we set CTC counts of 1, 2, 3, 4, and 5 as thresholds and found that patients with pre-surgical CTCs ≥ 4 had obviously but not significantly shorter DFS (p = 0.459) and OS (p = 0.209) than patients without CTC or patients with 1–3 CTCs (Additional file 4: Figure S2). The median DFS of patients without CTC, 1–3 CTCs, or more than 4 CTCs post-operatively were 33.87, 34.33, and 20.98 months respectively. As the threshold increased, we observed that pre-surgical CTCs ≥ 5 were significantly associated with worse outcomes (Fig. 3a–e). For this reason, we set 5 as the threshold value.

Since the prognostic value of pre-operative CTCs is likely to be affected by surgery, we suggest that post-operative CTCs might be a more direct and efficient recurrence marker than pre-operative CTCs. However, few reports have questioned whether monitoring post-operative CTCs is useful for predicting prognosis. Our results demonstrated that patients with post-operative CTCs ≥ 4–5 had shorter DFS, OS, and less 3-years DFS (Fig. 3a–e, Additional file 4: Figure S2). In addition, acting as the “seed” of hematogenous metastasis, CTCs can travel in the circulation and form metastases [9, 10]. High post-operative CTCs and markedly increased post-operative CTCs were correlated with hematogenous metastasis, while pre-operative CTCs were not correlated with recurrence patterns. Our results also confirmed that post-operative CTCs might be a more direct post-operative recurrence marker especially for patients who experienced hematogenous recurrence. As a result, monitoring the recurrence patterns of gastric cancer is important for better post-operative adjuvant treatment decisions.

There are several limitations in our study. First, our study aimed to analyze the clinical significance of CTCs in radical gastric cancer, and patients with stage I and II accounted for 51.6% of all of the patients. Furthermore, CTCs ≥ 3–5 were identified in a few patients. For future work, more subjects are needed to determine an optimal threshold, and a sufficient sample size to adequately perform Kaplan–Meier analyses. Second, mesenchymal-like cancer cells are likely to escape from CellSearch system detection, which is based on epithelial markers, such as EpCAM and CKs [17]. Nonetheless, in future studies, we can combine other methods of molecular analysis to better identify the prognostic value of CTCs in resectable gastric cancer. Third, numerous studies agree that intra-operative CTCs shedding occurs with tumor manipulation [24]. However, our data suggested that post-operative CTC enumeration within 1 week after surgery was not increased by surgery. Although intra-operative CTCs were not detected in our study, studies of other tumors reported that CTC detection rate and not CTC enumeration increased in intra-operative samples [24]. Moreover, studies also demonstrated that increased intra-operative CTCs normalize post-operatively within days to weeks [24]. Fourth, we collected post-operative samples within 1 week after surgery (2–7 days after surgery). We are also interested in how CTCs can change when the length of observation is increased to two or 3 weeks, which we will address in follow-up studies. Interestingly, Krag and colleagues reported that CTCs rapidly declined during 48 h post-operatively in most patients with operable breast cancer [25]. Animal studies also found the similar phenomenon [26]. Krag et al. also demonstrated that 30% patients had detectable CTCs persistently up to 14 days after surgery. These results are consistent with the notion that only a minority of CTCs can survive and have capacity to be clonogenic. In addition, those authors further put forward a view, which is similar to us, namely, the persistent presence of cancer cells after radical surgery is a strong indicator of recurrence. Therefore, we hypothesize that CTCs are stable from 2 days to 2 weeks, and CTCs should be monitored before and after surgery to predict cancer recurrence and for disease staging and treatment management.

## Conclusion

In summary, we analyzed the utility of post-operative CTCs in resectable gastric cancer. In addition, to the best of our knowledge, this is the first prospective study that has analyzed the correlations between post-operative CTCs and changes in CTCs during surgery with clinicopathological characteristics, prognosis, and recurrence patterns. We found that post-operative CTCs might be a more direct and efficient recurrence marker than pre-operative CTCs and increased post-operative CTCs might be correlated with hematogenous recurrence. Monitoring the post-operative CTCs and changes in CTCs during surgery is of great importance for better post-operative adjuvant treatment decisions.

## Additional files

**Additional file 1: Table S1.** Patient characteristics and CTC numbers in 63 patients.

**Additional file 2: Table S2.** Patient characteristics and CTC numbers in 31 relapsed patients.

**Additional file 3: Figure S1.** Correlations of pre-operative CTCs with patient recurrence patterns.

**Additional file 4: Figure S2.** Patients who have ≥ 4 CTCs/7.5 ml blood pre-operatively had shorter DFS (a, c) and OS (b, d).

## Abbreviations

CTC: circulating tumor cell
CEA: carcinoembryonic antigen
OS: overall survival
EpCAM: epithelial cell adhesion molecule
CK: cytokeratin
DAPI: 4′, 6-diamidino-2-phenylindole
DFS: disease-free survival
3-year DFS: 3-year disease-free survival rate
