# Supplementary material for: A prospective study on the changes and clinical significance of pre-operative and post-operative circulating tumor cells in resectable gastric cancer
Source: J Transl Med. 2018 Jun 20;16:171. doi: 10.1186/s12967-018-1544-1 (PMC6011408; doi:10.1186/s12967-018-1544-1)
Supplement: Supplementary file 1 — Additional file 1: Table S1. Patient characteristics and CTC numbers in 63 patients. [file 12967_2018_1544_MOESM1_ESM.doc]

**Table S1. Patient characteristics and CTC numbers in 63** patients

| **Patients Number** | **Gender** | **Age** | **Primary- site ^†^** | **Differentiation ^‡^** | **Lauren** | **TNM** | **Depth of Penetration** | **Lymph node** | **Lymph-vascular invasion** | **Pre-**  **Surgery**  **CTC** | **Post-**  **Surgery**  **CTC** |
| --- | --- | --- | --- | --- | --- | --- | --- | --- | --- | --- | --- |
| Patient 1 | Female | 31 | Non-EGJ | Poor | Mixed | I | T1 | N0 | NO | 0 | 0 |
| Patient 2 | Female | 61 | Non-EGJ | Poor | Diffuse | I | T1 | N0 | NO | 0 | 0 |
| Patient 3 | Male | 42 | Non-EGJ | Poor | Diffuse | I | T1 | N0 | NO | 0 | 0 |
| Patient 4 | Male | 42 | EGJ | Poor | Mixed | III | T4 | N3 | YES | 0 | 0 |
| Patient 5 | Male | 57 | Non-EGJ | Poor | Mixed | III | T3 | N2 | YES | 1 | 0 |
| Patient 6 | Female | 60 | Non-EGJ | Poor | Diffuse | III | T3 | N3 | YES | 0 | 0 |
| Patient 7 | Male | 48 | Non-EGJ | Good | Intestinal | I | T2 | N0 | NO | 0 | 0 |
| Patient 8 | Male | 56 | Non-EGJ | Poor | Diffuse | II | T2 | N1 | YES | 0 | 0 |
| Patient 9 | Male | 47 | Non-EGJ | Good | Intestinal | I | T1 | N0 | NO | 0 | 0 |
| Patient 10 | Female | 55 | Non-EGJ | Poor | Intestinal | III | T4 | N2 | NO | 0 | 0 |
| Patient 11 | Male | 66 | EGJ | Good | Intestinal | I | T1 | N0 | NO | 1 | 1 |
| Patient 12 | Male | 46 | Non-EGJ | Poor | Mixed | I | T1 | N0 | NO | 0 | 0 |
| Patient 13 | Male | 53 | EGJ | Poor | Mixed | II | T3 | N1 | YES | 1 | 0 |
| Patient 14 | Male | 70 | Non-EGJ | Good | Intestinal | III | T4 | N3 | YES | 1 | 0 |
| Patient 15 | Female | 56 | EGJ | Poor | Intestinal | II | T3 | N1 | YES | 0 | 0 |
| Patient 16 | Male | 70 | Non-EGJ | Poor | Mixed | III | T4 | N2 | NO | 5 | 1 |
| Patient 17 | Female | 41 | Non-EGJ | Poor | Mixed | I | T1 | N0 | NO | 1 | 1 |
| Patient 18 | Male | 62 | Non-EGJ | Poor | Mixed | III | T4 | N3 | YES | 0 | 0 |
| Patient 19 | Male | 67 | Non-EGJ | Good | Mixed | II | T2 | N1 | NO | 0 | 0 |
| Patient 20 | Male | 57 | Non-EGJ | Good | Intestinal | III | T3 | N3 | YES | 0 | 0 |
| Patient 21 | Male | 60 | Non-EGJ | Poor | Mixed | II | T3 | N1 | YES | 1 | 0 |
| Patient 22 | Male | 64 | EGJ | Poor | Intestinal | I | T1 | N0 | NO | 0 | 0 |
| Patient 23 | Male | 75 | EGJ | Poor | Mixed | II | T3 | N1 | YES | 0 | 1 |
| Patient 24 | Male | 67 | EGJ | Poor | Diffuse | III | T4 | N3 | YES | 67 | 96 |
| Patient 25 | Female | 39 | Non-EGJ | Poor | Diffuse | III | T3 | N3 | YES | 3 | 1 |
| Patient 27 | Female | 48 | Non-EGJ | Poor | Mixed | I | T1 | N1 | YES | 0 | 0 |
| Patient 28 | Male | 81 | Non-EGJ | Poor | Mixed | III | T4 | N1 | NO | 0 | 0 |
| Patient 30 | Male | 55 | Non-EGJ | Poor | Intestinal | III | T4 | N3 | YES | 0 | 0 |
| Patient 31 | Male | 58 | EGJ | Good | Intestinal | II | T3 | N1 | NO | 0 | 0 |
| Patient 33 | Male | 76 | Non-EGJ | Poor | Intestinal | III | T4 | N2 | YES | 0 | 3 |
| Patient 38 | Male | 67 | Non-EGJ | Poor | Mixed | III | T4 | N3 | YES | 1 | 7 |
| Patient 39 | Male | 58 | EGJ | Good | Intestinal | III | T4 | N2 | YES | 0 | 0 |
| Patient 41 | Male | 59 | EGJ | Good | Intestinal | III | T4 | N1 | NO | 0 | 4 |
| Patient 42 | Male | 50 | EGJ | Good | Intestinal | III | T4 | N3 | YES | 0 | 0 |
| Patient 44 | Male | 69 | Non-EGJ | Good | Intestinal | II | T3 | N0 | NO | 0 | 2 |
| Patient 45 | Male | 45 | Non-EGJ | Poor | Mixed | III | T4 | N3 | NO | 26 | 4 |
| Patient 46 | Male | 63 | Non-EGJ | Poor | Mixed | III | T4 | N3 | YES | 1 | 1 |
| Patient 47 | Female | 38 | Non-EGJ | Poor | Diffuse | III | T4 | N2 | NO | 0 | 8 |
| Patient 48 | Male | 57 | EGJ | Poor | Diffuse | III | T3 | N2 | NO | 0 | 4 |
| Patient 49 | Male | 56 | EGJ | Poor | Intestinal | III | T3 | N3 | NO | 1 | 0 |
| Patient 52 | Male | 70 | EGJ | Poor | Diffuse | III | T3 | N3 | YES | 0 | 0 |
| Patient 53 | Male | 63 | EGJ | Poor | Mixed | II | T3 | N1 | NO | 0 | 145 |
| Patient 57 | Female | 67 | EGJ | Poor | Intestinal | II | T3 | N1 | NO | 0 | 0 |
| Patient 58 | Female | 26 | Non-EGJ | Poor | Diffuse | III | T4 | N3 | YES | 3 | 0 |
| Patient 59 | Female | 64 | EGJ | Poor | Intestinal | III | T3 | N3 | YES | 1 | 0 |
| Patient 60 | Male | 60 | Non-EGJ | Poor | Diffuse | I | T2 | N0 | NO | 0 | 0 |
| Patient 61 | Male | 49 | Non-EGJ | Good | Intestinal | II | T4 | N0 | YES | 3 | 3 |
| Patient 62 | Female | 66 | Non-EGJ | Poor | Diffuse | II | T3 | N1 | YES | 0 | 0 |
| Patient 63 | Female | 66 | Non-EGJ | Good | Intestinal | II | T3 | N0 | NO | 0 | 0 |
| Patient 64 | Male | 36 | Non-EGJ | Poor | Diffuse | III | T3 | N2 | YES | 0 | 0 |
| Patient 67 | Male | 60 | Non-EGJ | Poor | Mixed | I | T2 | N0 | YES | 1 | 0 |
| Patient 75 | Male | 53 | Non-EGJ | Poor | Diffuse | II | T3 | N1 | YES | 2 | 4 |
| Patient 77 | Male | 41 | Non-EGJ | Poor | Mixed | III | T3 | N3 | YES | 0 | 1 |
| Patient 78 | Female | 50 | Non-EGJ | Poor | Diffuse | I | T1 | N0 | NO | 0 | 0 |
| Patient 79 | Female | 45 | Non-EGJ | Poor | Mixed | I | T1 | N1 | YES | 1 | 0 |
| Patient 83 | Male | 68 | Non-EGJ | Poor | Diffuse | II | T2 | N2 | YES | 85 | 0 |
| Patient 86 | Male | 80 | EGJ | Poor | Intestinal | III | T3 | N3 | NO | 0 | 1 |
| Patient 87 | Male | 72 | EGJ | Good | Intestinal | III | T3 | N3 | NO | 0 | 1 |
| Patient 88 | Male | 45 | Non-EGJ | Poor | Intestinal | I | T1 | N0 | NO | 0 | 0 |
| Patient 89 | Male | 57 | Non-EGJ | Poor | Diffuse | III | T3 | N2 | YES | 0 | 0 |
| Patient 90 | Male | 60 | Non-EGJ | Poor | Mixed | III | T3 | N3 | YES | 2 | 1 |
| Patient 91 | Male | 73 | Non-EGJ | Poor | Mixed | III | T4 | N3 | YES | 0 | 0 |
| Patient 92 | Female | 45 | Non-EGJ | Good | Intestinal | II | T1 | N2 | NO | 0 | 0 |

Note: † EGJ, gastroesophageal junction; ‡ Good, including high or moderate differentiation; Poor, including low differentiation, mucinous adenocarcinoma, and signet-ring cell carcinoma.
