# Supplementary material for: A prospective study on the changes and clinical significance of pre-operative and post-operative circulating tumor cells in resectable gastric cancer
Source: J Transl Med. 2018 Jun 20;16:171. doi: 10.1186/s12967-018-1544-1 (PMC6011408; doi:10.1186/s12967-018-1544-1)
Supplement: Supplementary file 2 — Additional file 2: Table S2. Patient characteristics and CTC numbers in 31 relapsed patients. [file 12967_2018_1544_MOESM2_ESM.doc]

**Table S2. Patient characteristics and CTC numbers in 31 relapsed patients**

| **Patients Number** | **Gender** | **Age** | **Primary site ^†^** | **Preoperative CTC count** | **Postoperative CTC count** | **DFS ^‡^ (months)** |
| --- | --- | --- | --- | --- | --- | --- |
| Patient 4 | Male | 42 | EGJ | 0 | 0 | 20.2 |
| Patient 8 | Male | 56 | Non-EGJ | 0 | 0 | 11.9 |
| Patient 10 | Female | 55 | Non-EGJ | 0 | 0 | 9.9 |
| Patient 14 | Male | 70 | Non-EGJ | 1 | 0 | 20.0 |
| Patient 15 | Female | 56 | EGJ | 0 | 0 | 5.17 |
| Patient 18 | Male | 62 | Non-EGJ | 0 | 0 | 14.8 |
| Patient 20 | Male | 57 | Non-EGJ | 0 | 0 | 5.97 |
| Patient 21 | Male | 60 | Non-EGJ | 1 | 0 | 15.5 |
| Patient 23 | Male | 75 | EGJ | 0 | 1 | 25.9 |
| Patient 24 | Male | 67 | EGJ | 67 | 96 | 1.13 |
| Patient 25 | Female | 39 | Non-EGJ | 3 | 1 | 28.4 |
| Patient 26 | Male | 63 | EGJ | 0 | — | 11.2 |
| Patient 28 | Male | 81 | Non-EGJ | 0 | 0 | 12.3 |
| Patient 32 | Male | 55 | Non-EGJ | 1 | — | 29.5 |
| Patient 36 | Male | 71 | EGJ | 0 | — | 11.0 |
| Patient 38 | Male | 67 | Non-EGJ | 1 | 7 | 1.13 |
| Patient 39 | Male | 58 | EGJ | 0 | 0 | 30.0 |
| Patient 45 | Male | 45 | Non-EGJ | 26 | 4 | 7.87 |
| Patient 48 | Male | 57 | EGJ | 0 | 4 | 31.4 |
| Patient 50 | Male | 72 | Non-EGJ | 0 | — | 6.5 |
| Patient 53 | Male | 63 | EGJ | 0 | 145 | 6.63 |
| Patient 57 | Female | 67 | EGJ | 0 | 0 | 31.6 |
| Patient 58 | Female | 26 | Non-EGJ | 3 | 0 | 15.5 |
| Patient 59 | Female | 64 | EGJ | 1 | 0 | 0.8 |
| Patient 68 | Female | 29 | Non-EGJ | 0 | — | 8.8 |
| Patient 76 | Female | 48 | Non-EGJ | 2 | — | 21.2 |
| Patient 81 | Male | 61 | Non-EGJ | 27 | — | 15.2 |
| Patient 87 | Male | 72 | EGJ | 0 | 1 | 4.9 |
| Patient 90 | Male | 60 | Non-EGJ | 2 | 1 | 11.3 |
| Patient 91 | Male | 73 | Non-EGJ | 0 | 0 | 20.7 |
| Patient 92 | Female | 45 | Non-EGJ | 0 | 0 | 21.8 |

Note: † EGJ, gastroesophageal junction; ‡ DFS, disease-free survival.
