# Supplementary figures and images for: A prospective study on the changes and clinical significance of pre-operative and post-operative circulating tumor cells in resectable gastric cancer
Source: J Transl Med. 2018 Jun 20;16:171. doi: 10.1186/s12967-018-1544-1 (PMC6011408; doi:10.1186/s12967-018-1544-1)

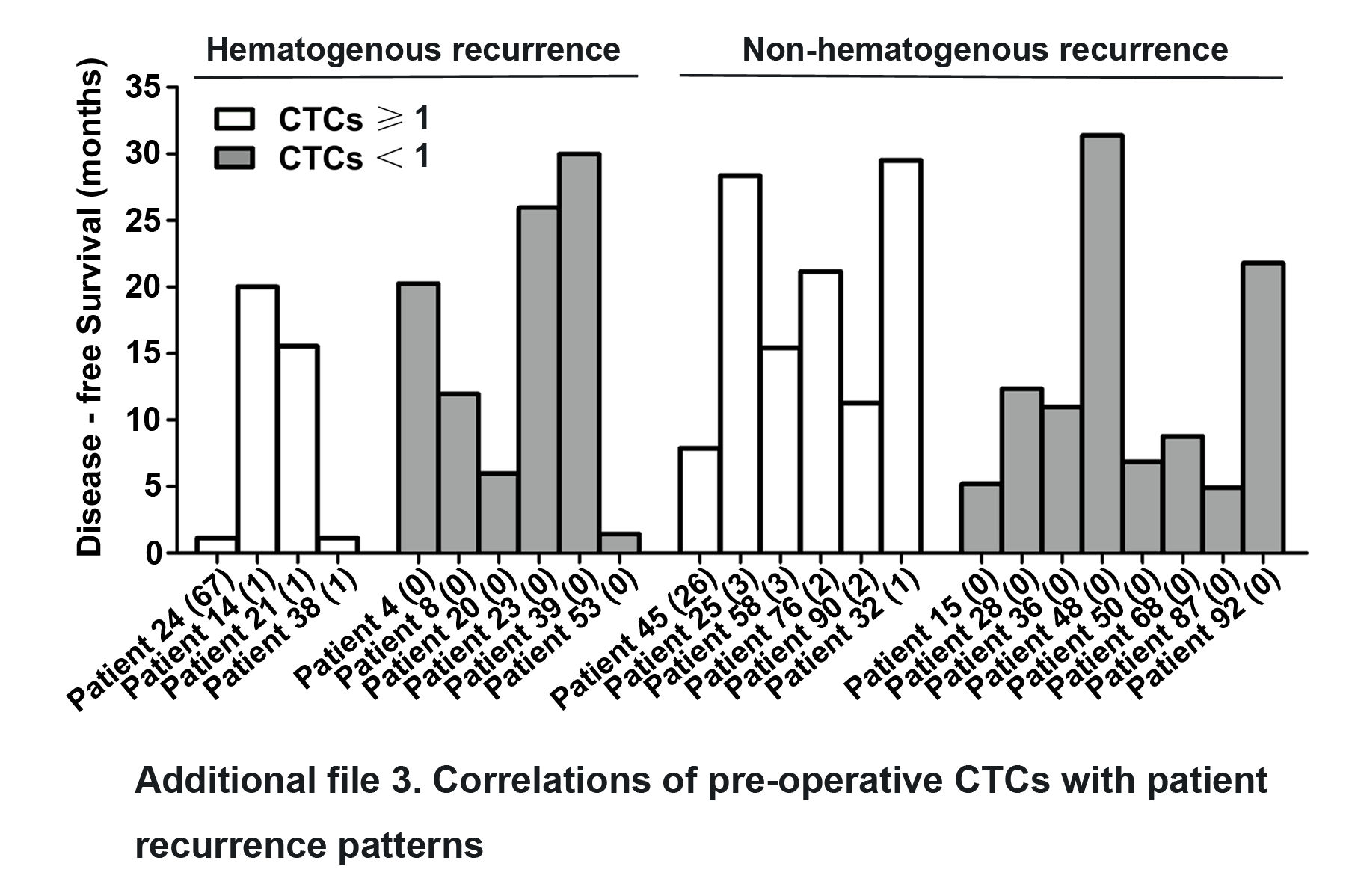

Supplement: Supplementary file 3 — Additional file 3: Figure S1. Correlations of pre-operative CTCs with patient recurrence patterns. [file 12967_2018_1544_MOESM3_ESM.tif]

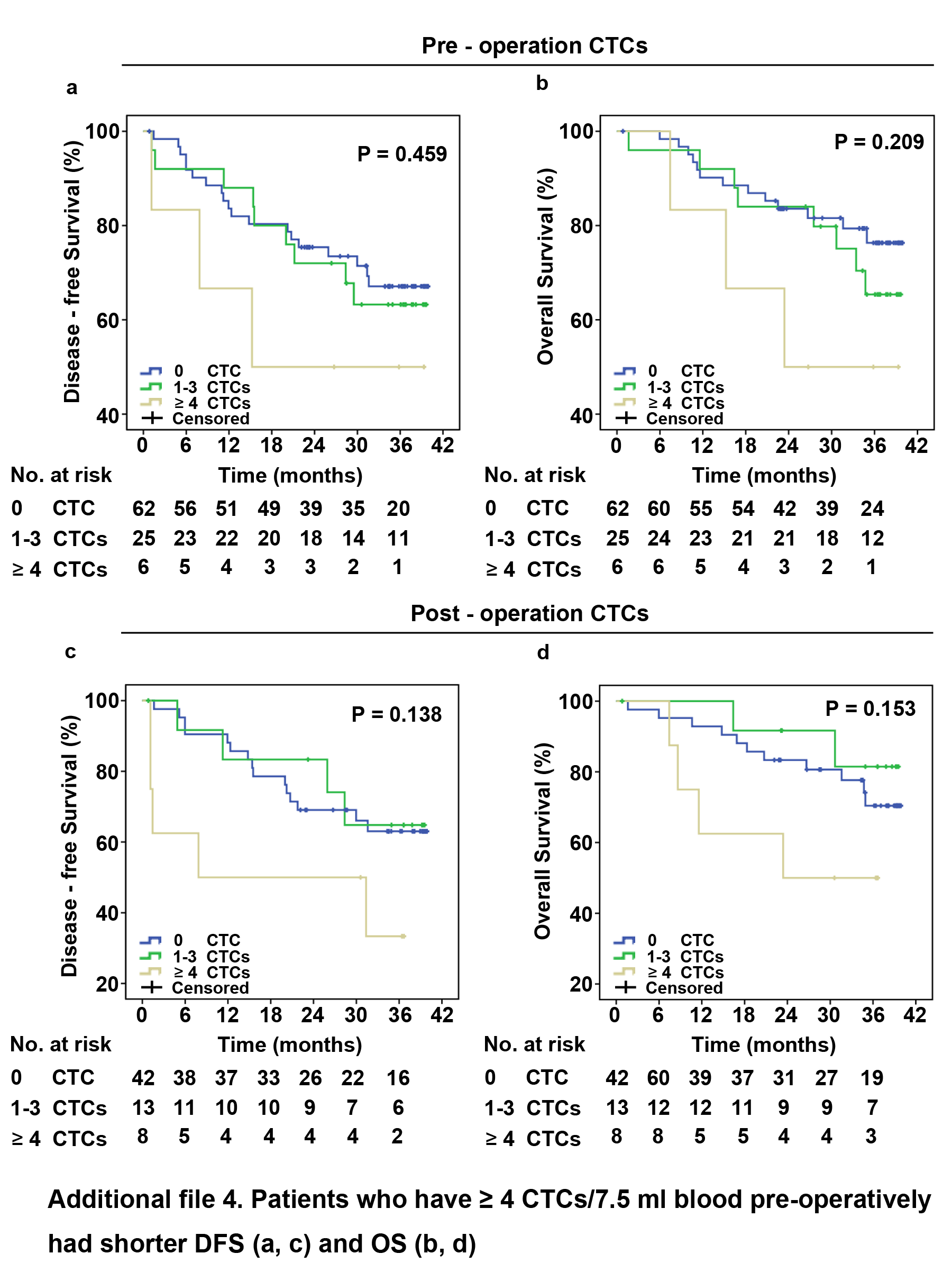

Supplement: Supplementary file 4 — Additional file 4: Figure S2. Patients who have ≥ 4 CTCs/7.5 ml blood pre-operatively had shorter DFS (a, c) and OS (b, d). [file 12967_2018_1544_MOESM4_ESM.tif]
